# Rare entities in head-and-neck cancer: salvage re-irradiation with carbon ions

**DOI:** 10.1186/s13014-019-1406-x

**Published:** 2019-11-12

**Authors:** Thomas Held, Paul Windisch, Sati Akbaba, Kristin Lang, Benjamin Farnia, Jakob Liermann, Denise Bernhardt, Peter Plinkert, Christian Freudlsperger, Stefan Rieken, Klaus Herfarth, Jürgen Debus, Sebastian Adeberg

**Affiliations:** 1 Department of Radiation Oncology, Heidelberg University Hospital, Heidelberg, Germany; 2 Heidelberg Institute of Radiation Oncology (HIRO), Heidelberg, Germany; 3 National Center for Tumor diseases (NCT), Heidelberg, Germany; 4 Department of Radiation Oncology, University of Miami, Miami, Florida USA; 5 Department of Otorhinolaryngology, University of Heidelberg, Heidelberg, Germany; 6 Department of Oral and Maxillofacial Surgery, University Hospital Heidelberg, Heidelberg, Germany; 7 Heidelberg Ion-Beam Therapy Center (HIT), Heidelberg, Germany; 8 Clinical Cooperation Unit Radiation Oncology, German Cancer Research Center (DKFZ), Heidelberg, Germany; 9 German Cancer Consortium (DKTK), partner site Heidelberg, German Cancer Research Center (DKFZ), Heidelberg, Germany

**Keywords:** Salvage re-irradiation, Head and neck cancer, Carbon ions, Mucoepidermoid carcinoma, Particle therapy

## Abstract

**Background:**

The objective of this investigation is to evaluate the outcomes and toxicity of carbon-ion re-irradiation (CIR) in patients with rare head and neck cancers (HNC). There is a paucity of data regarding treatment approaches in this patient cohort, which we aim to address in this work.

**Methods:**

Thirty-two (*n* = 32) consecutive patients with uncommon HNC treated between 2010 and 2017 were retrospectively analyzed in terms of clinical outcomes, patterns of failure, and toxicity.

**Results:**

Mucoepidermoid carcinoma (MEC) was the most common histology (22%). Patients received a median cumulative dose equivalent in 2 Gy fractions (EQD_2_) after CIR of 128.6 Gy (range, 105.8–146.5 Gy). The local and distant control rates 1 year after CIR were 66 and 72%. No serious acute or late toxicity (≥ grade 3) after CIR was observed.

**Conclusions:**

CIR may represent an effective and safe treatment alternative to palliative systemic therapies in these rare indications.

## Introduction

Local recurrence or tumor progression after multimodal therapy for head and neck cancer (HNC) occurs in up to 30–50% of patients [1, 2]. Treatment options for recurrent or progressive head and neck tumors are limited and generally associated with increased toxicities [3, 4]. Indeed, this cohort of patients is frequently studied in clinical trials [5, 6]. However, there are currently no guidelines for the clinical management of uncommon head and neck tumor entities, such as mucoepidermoid carcinoma (MEC), acinar cell carcinoma, and esthesioneuroblastoma.

Diagnostic uncertainties due either to small biopsy specimens or interpretive difficulties are common in patients with rare HNC entities, such as in the salivary glands [7]. Additionally, histopathological evaluation of head and neck malignancies may be subject to pronounced inter-observer variability [8]. Finally, several factors, such as the patient’s performance status, the time interval between treatments, and eligibility for surgical intervention, are critical when determining clinical management [9–11]. These challenges underscore the difficulty in treating this patient population.

Re-irradiation with photons has previously been described in patients with squamous cell carcinoma (SCC) of the head and neck and was found not only to be associated with poor clinical outcomes but also with high treatment morbidity, particularly when combined with chemotherapy [1, 2]. In inoperable situations, re-irradiation with heavy ions has previously been described as feasible and effective in patients with recurrent nasopharyngeal HNC [12]. For patients with recurrent adenoid cystic carcinoma commonly treated with carbon ions at our clinic, encouraging results regarding toxicity and local control have been reported [13]. The rationale for heavy particle radiation with high linear energy transfer (LET) is an increase in relative biological effectiveness (RBE) compared to photons with improved physical depth-dose distributions, effectively reducing the dose delivered to adjacent normal tissues while simultaneously allowing dose escalation in the recurrent tumor [14, 15].

In summary, diagnostic uncertainties and the absence of clinical guidelines impede treatment for uncommon tumor entities in recurrent HNC. The objective of this study is to investigate carbon-ion re-irradiation (CIR) treatment to address this paucity of data.

## Methods

### Patient characteristics

Following approval by the local ethics committee, screening was conducted using our clinic’s cancer registry, which currently contains the records of over 3500 patients diagnosed with HNC. Patients were eligible for study inclusion if they had at least one prior radiotherapy (RT) treatment for HNC, were subsequently diagnosed with locally recurrent disease or tumor progression, and received CIR at our institution. At our clinic, the vast majority of patients presented with SCC followed by adenoid cystic carcinoma, which were not included in the current evaluation. Furthermore, patients with plasmocytoma, lymphoma, sarcoma, and chordoma were also excluded. Thirty-two (*n* = 32) consecutive patients with uncommon head and neck cancer entities treated at our clinic between 2010 and 2017 were retrospectively analyzed in terms of clinical outcomes, patterns of failure, and toxicity.

### Treatment planning and follow-up

Patients were immobilized with a thermoplastic head–mask system. Contrast-enhanced computed tomography (CT) scans (3-mm slice thickness) were used for treatment planning, and contrast-enhanced T1-weighted magnetic resonance imaging (MRI) was used for image registration. Treatment planning was conducted using Syngo PT Planning version 13 (Siemens®, Erlangen, Germany). The clinical target volume (CTV) included the visible tumor on contrast-enhanced CT or MRI (gross tumor volume or GTV) with a margin of 2–5 mm for subclinical disease spread. The resection cavity was included in the CTV for patients with prior surgical resection. Depending on patient positioning and beam arrangement, an additional margin of 2–3 mm was added for the planning target volume (PTV).

Treatment was exclusively performed with carbon ions using the active raster-scanning method with daily image guidance by orthogonal X-rays. Imaging follow-up included a contrast-enhanced MRI or a CT scan of the head and neck 6–8 weeks after treatment completion and every 3 months within the first 2 years after CIR. Treatment-related toxicities were recorded with the same frequency during follow-up visits and examinations by a radiation oncologist at our institution. Furthermore, patients saw an ear, nose, and throat (ENT) specialist at each follow-up visit.

### Statistics

Statistical analysis was conducted using SPSS Statistics 25 (IBM®, New York, USA) and the software R version 3.4.3 (www.r-project.org). The median follow-up for overall survival (OS) was calculated using the inverse Kaplan-Meier method. Local control (LC) was evaluated from the time interval between the start of CIR to the first occurrence of local failure using the Response Evaluation Criteria in Solid Tumors (RECIST) [16]. Similarly, local and distant progression-free survival (L-PFS, D-PFS) were measured from the time of CIR start to the first occurrence of local or distant failure, respectively. OS and progression-free survival (PFS) were calculated using the Kaplan-Meier method. OS was determined from the commencement of re-irradiation until death or last follow-up, whichever occurred first. Kaplan-Meier estimates for OS and PFS were tested for prognostic significance using the log-rank test and stepwise testing for significant cut-off values. The characteristics total dose of CIR (≥ 51 Gy (RBE) vs. < 51 Gy (RBE)), tumor histology (MEC vs. other), RT interval (≥ 2 years vs. < 2 years), distant metastatic spread (yes vs. no), and prior surgical resection (adjuvant vs. definitive CIR) were evaluated. Adverse events related to CIR were evaluated based on the patients’ medical records according to version 4.03 of the Common Terminology Criteria for Adverse Events (CTCAE).

## Results

### Tumor characteristics

**Fig. 1:**
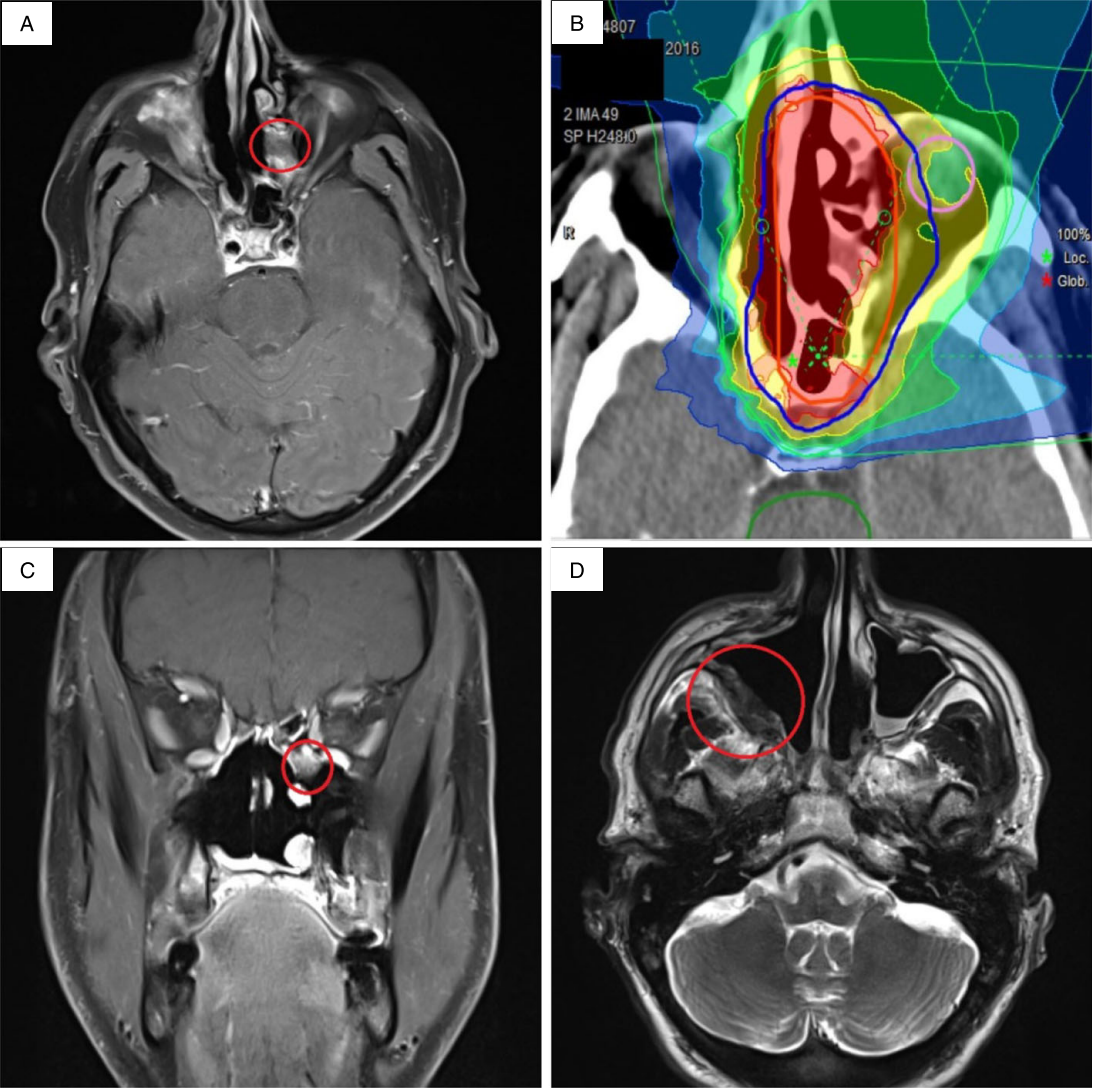
Shown are treatment and follow-up images of a 54 year old male patient with a recurrent small cell neuroendocrine carcinoma of the left paranasal sinus. After initial radiation treatment (66 Gy in 2 Gy fractions intensity-modulated radiation therapy) and three cycles of carboplatin and etoposide, the patient received re-irradiation with carbon ions (51 Gy (RBE) in 17 fractions) to the left paranasal sinus. The treatment planning T1 contrast-enhanced fat-suppressed magnetic resonance imaging (MRI) and the corresponding carbon ion treatment plan are shown in (**a**) and (**b**), respectively. The recurrent tumor was stable 24 months after treatment (**c**). Another 6 months later (30 months post-treatment), a recurrence developed in the contralateral paranasal sinus, shown as a T2w-hypointense tumor in the follow-up MRI (**d**)

Staging was conducted prior to CIR using the eighth edition of the Union for International Cancer Control (UICC) tumor-node-metastasis (TNM) classification. The majority of recurrent tumors were of an advanced stage (T3/T4, *n* = 24, 88.9%). Only four patients (*n* = 4, 12.5%) had distant metastasis prior to CIR, most commonly in the lungs (*n* = 3, 75.0%). The majority of recurrent tumors were localized in the major salivary glands (*n* = 15, 46.9%), the nasopharynx (*n* = 7, 21.9%), and the paranasal sinuses (*n* = 6, 18.8%). The majority of tumors were classified prior to re-irradiation as MEC (*n* = 7, 21.9%), acinar cell carcinoma (*n* = 6, 18.8%), esthesioneuroblastoma (*n* = 5, 15.6%), and myoepithelial carcinoma (*n* = 3, 9.4%). One patient had small cell neuroendocrine carcinoma (Fig. 1). Seven patients (*n* = 7, 21.9%) underwent surgical resection prior to CIR. Recurrence was pathologically confirmed for the majority of patients (*n* = 18, 56.3%), but the recurrent tumor was inaccessible for biopsy in seven patients (*n* = 7, 21.9%) and was defined radiographically. Among those undergoing pathological confirmation, in four patients (*n* = 4, 12.5%) histology was different compared with that at primary diagnosis.

### Treatment features

The median total dose of re-irradiation with carbon ions was 51 Gy (RBE) (range, 36–66 Gy (RBE)) with 3 Gy (RBE) per fraction in 5–6 fractions per week. None of the patients received simultaneous systemic therapies (e.g., chemotherapy) during CIR. On average, patients received four (range, 1–6) tumor-specific treatments (i.e., chemotherapy, surgery, or radiation treatment) before CIR. The median RT interval (i.e., time between initial RT and CIR) was 5.2 years (range, 0.6–46.5 years).

The initial RT was performed with 3D conformal RT in 12 patients (37.5%), intensity-modulated RT (IMRT) in 9 patients (28.1%), bimodal RT (IMRT and carbon ion boost) in 4 patients (12.5%), and other modalities (i.e., stereotactic body RT, cobalt therapy, or electron beam therapy) in 5 patients (15.6%) but was unknown in 2 patients (6.3%). Patients with bimodal RT received a median dose of 50 Gy (range, 50–54 Gy) with photon IMRT and a median dose of 24 Gy (RBE) (range, 18–24 Gy (RBE)) with carbon ions. The CTV and PTV of re-irradiation were 98.3 cubic cm (range, 13.3–550.6 cubic cm) and 137.1 cubic cm (range, 23.1–714.9 cubic cm), respectively. The median total tumor lifetime dose (equivalent in 2 Gy fractions, EQD2) after initial RT and CIR was 128.6 Gy (range, 105.8–146.5 Gy). An alpha-beta ratio of 10 Gy was used for all head and neck tumor entities, as previously reported [17]. Detailed patient and treatment characteristics are shown in Table 1.

**Table 1:** Patient and treatment characteristics (*n* = 32 patients)

Patient characteristics		
	Patients	%
Gender		
Female	15	46.9
Male	17	53.1
Age		
≥ 60 years	19	59.4
< 60 years	12	40.6
ECOG status		
0	16	50.0
1	16	50.0
Histology		
Mucoepidermoid carcinoma	7	21.9
Acinar cell carcinoma	6	18.8
Esthesioneuroblastoma	5	15.6
Lymphoepithelial carcinoma	4	12.5
Myoepithelial carcinoma	3	9.4
Sinonasal undifferentiated carcinoma	2	6.2
Salivary duct carcinoma	2	6.2
Other	3	9.4
Tumor site		
Major salivary gland	15	46.8
Nasopharynx	7	21.9
Paranasal sinus	6	18.8
Other	4	12.5
Tumor stage		
T1 + T2	3	9.4
T3 + T4	24	75.0
Undetermined	5	15.6
Metastasis stage		
M0	28	87.5
M1	4	12.5
Treatment characteristics		
Salvage surgery		
No	25	78.1
Yes	7	21.9
Re-irradiation		
	Median	Range
Total dose CIR [Gy (RBE)]	51.0	36.0–66.0
Cumulative tumor lifetime dose [EQD2]	128.6	105.8–146.5
CTV re-irradiation [cubic cm]	98.3	13.3–550.6
PTV re-irradiation [cubic cm]	137.1	23.1–714.9

Abbreviations: Eastern Cooperative Oncology Group (ECOG), carbon ion re-irradiation (CIR), Gray (Gy), clinical target volume (CTV), planning target volume (PTV), equivalent dose in 2 Gy fractions (EQD2), radiotherapy (RT)

### Acute and late toxicity

The median follow-up interval after CIR was 18.1 months (range, 3.2–51.2 months). Almost all patients completed re-irradiation without interruption (*n* = 31, 96.9%). Treatment was cancelled for one patient due to newly diagnosed leptomeningeal spread. No severe acute (during the initial 90 days after CIR) or late treatment-related toxicities (≥ grade III) were observed after CIR. The most common low-grade acute toxicities included grade I and grade II (*n* = 12 and 1, 37.5 and 3.1%) radiation dermatitis and grade I and grade II oral mucositis (*n* = 4 and 4, 12.5 and 12.5%). Late treatment-related toxicities could be evaluated in 21 patients (*n* = 21, 65.6%) with available follow-up imaging and clinical data. Common low-grade late toxicities included grade I and grade II middle ear inflammation (*n* = 2 and 3, 9.5 and 14.3%), grade II dysgeusia (*n* = 3, 14.3%), and grade I and grade II (*n* = 2 and 3, 9.5 and 14.3%) hearing impairment. One patient developed a symptomatic blood–brain barrier disruption in the left temporal lobe 13.5 months after CIR that was treated with oral dexamethasone. Another patient with an MEC of the right cavernous sinus developed a brief generalized seizure, possibly related to CIR. Detailed information on acute and late treatment-related toxicities is shown in Table 2.

**Table 2:** Acute and late treatment related toxicity

	Patients	%
Acute toxicity		
Grade II		
Oral mucositis	4	12.5
Dysphagia	2	6.2
Grade I		
Radiation dermatitis	12	37.5
Xerostomia	7	21.9
Oral mucositis	4	12.5
Hearing impairment	3	9.4
Late toxicity		
Grade II		
Hearing impairment	3	14.3
Middle ear inflammation	3	14.3
Trigeminal nerve disorder	2	9.5
CNS necrosis	1	4.8
Dysphagia	1	4.8
Grade I		
Trismus	3	14.3
Facial edema	2	9.5
Middle ear inflammation	2	9.5
Dysgeusia	2	9.5
Xerostomia	2	9.5
Hearing impairment	2	9.5

### Clinical outcome and patterns of failure

The median local progression-free (L-PFS) survival was 24.2 months (95% CI, 21.0–27.5 months). Patients with a total dose of CIR ≥ 51 Gy (RBE) had a significantly superior local L-PFS of 31.8 months compared to 5.1 months (*p* = 0.001), independent of tumor histology. The local and distant control 1 year after CIR was 66 and 72%, respectively. The majority of local recurrences (87.5%) after CIR were in-field.

Of all patients with progressive disease (PD) after CIR, 15 (68.2%) failed locally and seven (*n* = 7, 31.8%) failed distantly. The pattern of failure was defined according to the site of first failure.

**Fig. 2:**
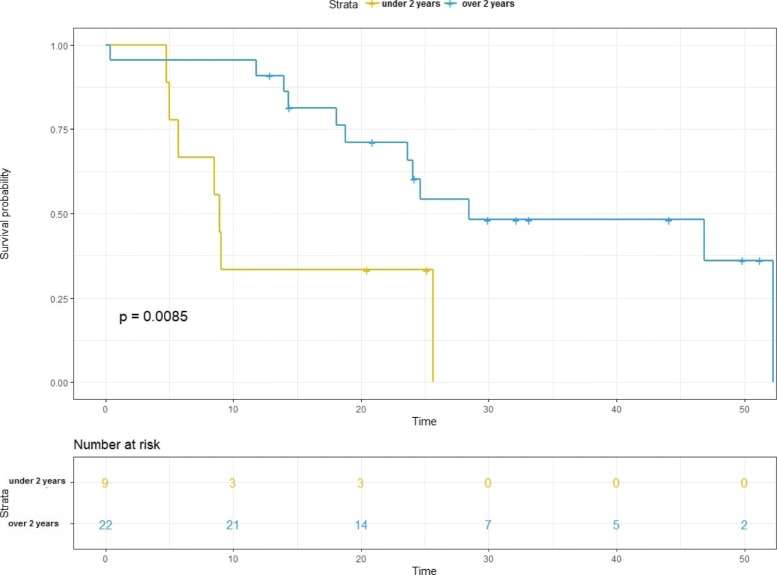
The median overall survival after carbon ion re-irradiation (CIR) in patients with recurrent head-and-neck cancer (HNC) was 28.5 months for patients with a radiotherapy (RT) interval ≥ 2 years compared to 8.9 months for patients with a RT interval < 2 years (*p* = 0.001)

The median OS was 24.7 months (95% CI, 21.9–27.5 months). The median OS after CIR was 28.5 months for patients with an RT interval ≥ 2 years compared to 8.9 months for patients with an RT interval < 2 years (*p* = 0.001; Fig. 2). The 6-, 12- and 18-month OS after CIR was 87.1, 77.4, and 61.3%, respectively. In patients with metastatic disease prior to re-irradiation (*n* = 4, 12.5%) there was no significant difference regarding OS within the first 2 years after CIR (24.1 months vs. 24.7 months, *p* = 0.995). Patients with two prior irradiation treatments compared to one prior course of radiation therapy had a significantly worse OS (4.8 months vs. 25.6 months, *p* = 0.001). There was no difference in OS for patients who underwent definitive (*n* = 25, 78.1%) compared to adjuvant (*n* = 7, 21.9%) CIR (28.5 months vs. 24.1 months, *p* = 0.388). In addition, patients with MEC had a worse OS compared to other histologies but without statistical significance (18.1 months vs. 25.6 months, *p* = 0.225). A total of 14 patients (*n* = 14, 43.8%) survived at least 2 years after CIR.

## Discussion

In the current analysis, we investigated salvage RT with carbon ions in 32 patients with uncommon biological entities of recurrent HNC. There are no treatment guidelines concerning the clinical management of recurrent head-and-neck tumors for these rare indications. The outcomes in our investigation were encouraging, with a 1-year local control of 66% in patients with biologically diverse yet unfavorable tumor features. In addition, patients who received a total dose of CR ≥ 51 Gy (RBE) had a significantly better local L-PFS, emphasizing the effects of dose escalation in recurrent HNC.

MECs, comparably frequent malignancies of the salivary glands, are known to develop local recurrence, particularly in cases of advanced T-stage, intermediate, or high-grade tumors [18]. In our analysis, we identified seven patients with recurrent MEC, with tumors categorized as intermediate or high grade in four patients (57%), low grade in one patient (14%), and undetermined in two patients (29%). Five patients (71%) had advanced T-stage tumors (T3/T4). An effective local salvage treatment for these rare indications is warranted in the case of recurrent disease, particularly in inoperable situations.

In our study, repeated acquisition of tumor biopsy specimens prior to CIR was not possible in seven patients (22%), mostly due to inaccessible localization of the recurrent cancer. Consequently, diagnostic uncertainties remain if biopsy acquisition is not feasible or is unsuccessful. Even when tissue specimens are available, morphologic similarity and overlapping immunohistochemical profiles [7] further impede correct diagnosis. The recurrent tumors of four patients (12.5%) in our cohort were re-classified to another histologic subtype when compared to the initial pathology report at diagnosis. The significance of this cannot be overstated given the variety of biological behaviors of each histologic subtype of HNC and the corresponding variations in treatment planning.

The feasibility of re-irradiation with heavy particles in patients with recurrent HNC has been documented in several previous studies [12, 13]. In our analysis, no severe (≥ grade III) treatment-related acute or late toxicities were observed. These findings are in line with an investigation on salvage irradiation with carbon ions in recurrent HNC that showed no acute toxicity and a 10% risk of severe late toxicity [19]. Compared to photon re-irradiation, which has a reported 30–50% rate of late toxicity ≥ grade III [4, 20], CIR appears favorable in this setting, with no severe acute or late toxicity identified. Consequently, our results underscore the use of heavy particle irradiation in mitigating treatment-related toxicity in this highly pretreated, vulnerable patient group.

Indeed, vital organs at risk, including the brain stem, optic system, and spinal cord, can be effectively spared due to the inverted depth-dose profile of carbon ion radiation [15]. This physical trait of heavy ions enables dose escalation to treat the tumor while reducing the dose to the previously irradiated, adjacent normal tissue. The RBE of carbon ions is higher compared to photons and protons [21], but the treatment effects in rare tumor entities and the implications for the clinical outcomes remain uncertain. In addition, the alpha/beta values of various histological entities may range from 2 to 10 Gy, thus impeding direct comparisons. Given that the RBE of carbon ions varies significantly for different biological and physical properties, uncertainties remain, and further clinical investigations are warranted.

In the current assessment, local control was non-inferior in patients who underwent definitive re-irradiation compared to patients who underwent a salvage operation prior to CIR. However, a difference was observed in the total dose delivered, with those in the definitive setting receiving a total dose ≥51 Gy (RBE) (*n* = 23) compared to adjuvant re-irradiation (*n* = 5), although without statistical significance (*p* = 0.201). The GTVs prior to the salvage operation or re-irradiation were comparable between both groups. While surgical resection should be considered in eligible patients, definitive re-irradiation appears non-inferior for patients with rare tumor entities of recurrent HNC.

Salvage re-irradiation is associated with distinct treatment-related toxicity due to the increased cumulative dose, highlighting the importance of adequate patient selection. In our assessment, patients who underwent two prior courses of radiation treatment showed a significantly inferior clinical outcome compared to patients with one prior course of irradiation (*p* = 0.001). Patients with MEC, particularly at an advanced stage and of intermediate or high grade, showed an inferior OS compared to other tumor entities but without statistical significance (*p* = 0.225). Beyond tumor histology and the number of irradiation treatments, additional aspects to be considered when selecting appropriate candidates include RT time intervals [11], previous irradiation fields (with particular attention to high-dose regions), and the patient’s performance status.

Several limitations of our investigation must be mentioned. A median follow-up interval of 18.1 months, in part due to the poor prognosis of patients with recurrent HNC, is considerably short to evaluate late treatment-related side effects. Additionally, our analysis is retrospective in nature, and information regarding adverse events was obtained from the patients’ medical records and thus subject to inherent bias. Furthermore, inaccuracies regarding the RBE of carbon ions in neoplasms with diverse biological features remain. Finally, the variety of tumor entities, each with unique biological behaviors, limits the applicability of our findings. However, given the paucity of data on these rare histologies and the corresponding lack of clinical trials, our findings represent an important contribution to the literature.

## Conclusions

Diagnostic uncertainties and the absence of treatment guidelines impede the clinical management of uncommon tumor entities in recurrent HNC. CIR may represent an effective and safe treatment alternative to surgical salvage, photon radiotherapy and palliative systemic therapies in these rare indications.

## Data Availability

All data generated or analyzed during this study are included in this published article.
